# Circulating Tumor DNA as Emerging Predictive and Prognostic Biomarker in Prostate Cancer

**DOI:** 10.3390/cancers18111702

**Published:** 2026-05-23

**Authors:** Bicky Thapa, Jacopo Venturini, Atish D. Choudhury, Edoardo Francini

**Affiliations:** 1 Lank Center for Genitourinary Oncology, Dana-Farber Cancer Institute, Boston, MA 02215, USA; bicky_thapa@dfci.harvard.edu (B.T.); atish_choudhury@dfci.harvard.edu (A.D.C.); 2 Sarah Cannon Research Institute (SCRI), London W1G 6AD, UK; drventurinij@gmail.com; 3 Department of Experimental and Clinical Medicine, University of Florence, 50134 Florence, Italy

**Keywords:** ctDNA, prostate cancer, NGS, precision medicine, epigenomics, androgen receptor pathway

## Abstract

A circulating tumor DNA (ctDNA) assay is an emerging non-invasive diagnostic approach providing real-time insights into the heterogeneous tumor molecular landscape of advanced prostate cancer, overcoming the limitations of traditional tissue biopsies and PSA. Detection methods include droplet digital PCR, next-generation sequencing, and new epigenomic and fragmentomic strategies (investigational) designed to improve sensitivity in cases of low ctDNA shedding. While ctDNA’s role in localized prostate cancer is limited, it offers significant prognostic value in metastatic cases, where high ctDNA levels correlate with shorter survival. Additionally, longitudinal ctDNA monitoring can predict treatment response and identify emerging resistance mechanisms, including androgen receptor alterations associated with androgen receptor pathway inhibitor therapy and BRCA reversion mutations linked to PARP inhibitors. Importantly, liquid biopsy enables genomic characterization to inform treatment decision-making, particularly in clinical scenarios where tissue biopsy is challenging, such as bone-only disease. However, the widespread clinical implementation of ctDNA analysis is hindered by several analytical challenges, including low sensitivity in localized disease and low disease burden, and the risk of false positives due to clonal hematopoiesis. Furthermore, greater efforts are required to standardize pre-analytical workflows and post-analytical data interpretation and reporting across institutions. This review aims to summarize the evolving role of cfDNA technologies in localized and advanced prostate cancer, highlighting their prognostic and predictive value and their role in uncovering mechanisms of treatment resistance.

## Simple Summary

Circulating tumor DNA (ctDNA) consists of small fragments of cancer DNA found in the bloodstream. In prostate cancer, ctDNA testing can give a “real-time” picture of the cancer’s genes without needing repeat tissue biopsies and adds information beyond the PSA blood test. In advanced prostate cancer, higher amounts of ctDNA are associated with shorter survival, and changes in ctDNA over time can show whether treatment is working or failing. ctDNA analysis can also find gene changes that help choose targeted treatments, such as BRCA mutations for PARP inhibitors or MSI-high status for immunotherapy. Additionally, tracking changes in ctDNA over time can reveal how cancer evolves and develops resistance to treatments. However, the utility of ctDNA in prostate cancer is still evolving, and limitations include low signal in early or low-volume disease, technical variability between labs, and “false” mutations from aging blood cells, so careful testing and standardization are needed.

## 1. Introduction

Prostate cancer is currently the second most commonly diagnosed cancer worldwide, with approximately 2.4 million cases projected by 2040 [1]. It is also the fifth leading cause of mortality among males [2]. Localized and locally advanced prostate cancer are associated with favorable outcomes owing to multimodal treatment strategies, including radical prostatectomy, radiation therapy, and hormone therapy.

Currently, treatment with androgen receptor pathway inhibitors (ARPIs) in combination with androgen deprivation therapy (ADT) is the standard of care for metastatic hormone-sensitive prostate cancer (mHSPC) and shows a median overall survival (OS) ranging between 4 and 8 years [3,4,5,6,7]. On the other hand, the median OS in metastatic castration-resistant prostate cancer (mCRPC) is around 2 years [8,9].

Prostate-specific antigen (PSA) is widely used for disease monitoring, often together with imaging techniques such as computed tomography (CT) scans, bone scans, and prostate-specific membrane antigen (PSMA) positron emission tomography (PET) scans [7].

In advanced prostate cancer, metastatic lesions harbor non-overlapping mutations, reflecting subclonal diversity arising during disease progression and under treatment pressure [10,11,12]. Consequently, comprehensive genomic profiling of single metastatic sites fails to capture the heterogeneous molecular landscape of the tumor, posing challenges for treatment effectiveness, particularly in mCRPC. Moreover, PSA reliability for monitoring disease progression may be limited in cases of low PSA release, such as in dedifferentiated disease [13,14,15]. These factors underscore the need for novel biomarkers capable of monitoring tumor evolution and identifying emerging resistant clones and actionable alterations. Non-invasive liquid biopsy methods have the potential to address these technical challenges, offering a real-time and dynamic depiction of the tumor’s molecular landscape. Cell-free DNA (cfDNA) is naturally found in the blood plasma and bodily fluids of both healthy individuals and those diagnosed with cancer. In healthy individuals, cfDNA arises from normal physiological processes, such as apoptosis, necrosis, inflammation, and secretion [16]. In contrast, circulating tumor DNA (ctDNA) represents the fraction of tumor-derived DNA released into the bloodstream by cancer cells through apoptosis, necrosis, and other mechanisms [16,17]. A growing body of evidence has investigated the role of ctDNA in prostate cancer across different disease settings, addressing its predictive and prognostic value [18,19]. Localized prostate cancer usually sheds very low quantities of ctDNA, whereas advanced prostate cancer typically displays higher shedding capacity [20,21,22]. ctDNA analysis in prostate cancer is an invaluable tool for the detection of clinically significant genomic mutations, copy number variations, structural rearrangements, and epigenomic changes [23]. Furthermore, ctDNA enables real-time assessment of tumor molecular heterogeneity to inform therapeutic decision-making, revealing the emergence of alterations associated with resistance or more aggressive phenotypes, such as TP53, RB1, PTEN, and BRCA1/2 [24,25]. Recent works have evaluated the clinical utility of ctDNA in prostate cancer from specific perspectives, including its prognostic significance in advanced disease, its role in early detection and disease monitoring, or its application in precision medicine for therapy selection [18,19,22,26]. In this review, we provide a more comprehensive overview of the evolving landscape of cfDNA technologies, analyzing in detail their clinical utility in localized and advanced prostate cancer, with a focus on both their prognostic and also predictive value. We further discuss the role of ctDNA in informing treatment decision-making by capturing tumor heterogeneity, emphasizing the molecular mechanisms of treatment resistance and tumor aggressiveness, as well as the current challenges that limit its clinical implementation.

## 2. Detection Methods and Technologies for Liquid Biopsies

**Figure 1:**
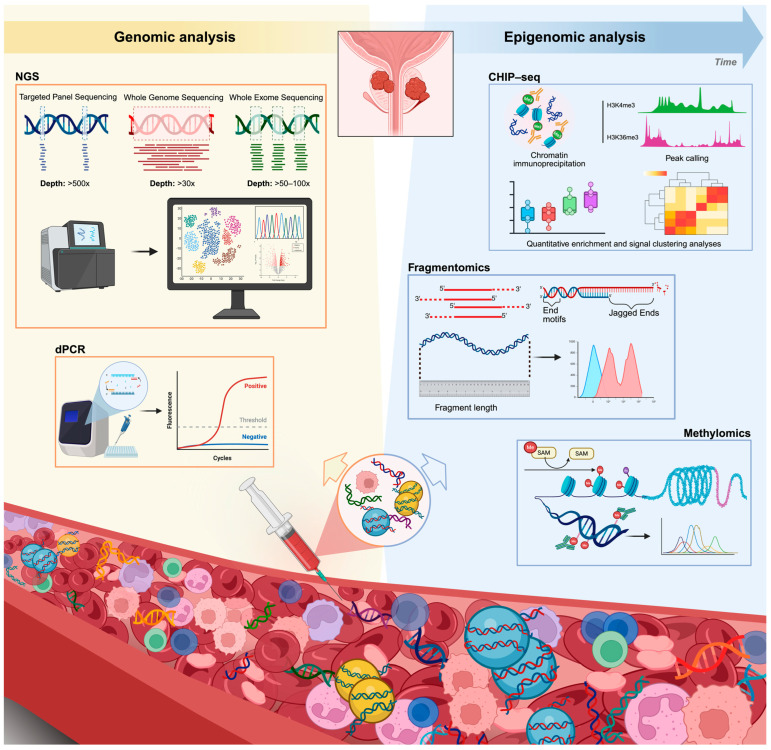
Epigenomic approaches such as methylomics, fragmentomics, and chromatin immunoprecipitation-based assays are third-generation cfDNA detection technologies that capture structural and regulatory signals, including fragment length, methylation patterns, nucleosome footprints, and histone modifications. Leveraging these features may overcome the sensitivity limits of genomic-based methods, thus broadening the scalability of liquid biopsy. Created in BioRender. Venturini, J. (2026) https://BioRender.com/fdfdk48.

A major technological challenge in liquid biopsy is the accurate detection of the minimal ctDNA fraction within a high background of normal cfDNA [27]. To address this, ctDNA detection techniques have undergone key advances over the past decade, with three distinct generations: the older two are referred to as “digital” and the most recent one as “analog”. Digital strategies detect discrete, predefined genomic alterations at specific loci, whereas analog strategies capture continuous genome-wide signals reflecting epigenomic patterns and chromatin organization [27] (Figure 1). Digital analytical assays are classified as targeted or untargeted. Targeted approaches are tumor-informed, as they track only predefined mutations derived from prior tissue sequencing analyses. This methodology is highly sensitive, detecting alterations at ultra-low variant allele frequencies (VAFs) [28]. Conversely, untargeted detection approaches use tumor-naïve assays. Although less sensitive than targeted assays, they enable plasma-only profiling when tissue is unavailable and allow discovery of novel and acquired alterations [29]. First-generation strategies consist of targeted, polymerase chain reaction (PCR)-based approaches which evolved from quantitative methods (i.e., qPCR) into the more clinically suitable digital formats such as droplet digital PCR (ddPCR) and Beads, Emulsion, Amplification, and Magnetics (BEAMing) [29]. ddPCR increases traditional qPCR sensitivity by 10–100-fold by partitioning samples into thousands of water-in-oil droplets, each acting as an independent PCR reactor [30,31]. BEAMing integrates emulsion PCR with magnetic beads and flow cytometry detection, maintaining ultra-high sensitivity (up to 0.01% VAF) [32]. Owing to their high analytical yield and cost-effectiveness, these methods are best suited to identify restricted numbers of hotspot mutations, such as for minimal residual disease (MRD) detection or tracking specific resistance variants in advanced disease settings. For instance, the androgen receptor (AR) splice variant 7 (AR-V7) has been associated with resistance to ARPIs for mCRPC [33,34]. This has been confirmed using ddPCR-based assays, with reported analytical sensitivity of 95–100% and specificity of 100% across 13 AR targets, including amplification, hotspot mutations, and splice variants [35].

Second-generation targeted ctDNA strategies use next-generation sequencing (NGS). Using capture probes, this approach enables parallel sequencing of multiple genes. The alignment to a reference genome then allows the simultaneous detection of different mutations, including low-VAF variants [36]. NGS strategies rely on amplicon- and capture-based methods [28]. Amplicon-based NGS assays (e.g., Safe-Sequencing System (Safe-SeqS)) leverage PCR amplifications of predefined genomic regions (termed “amplicons”), generating libraries of short amplicons that are subsequently sequenced in parallel [29]. However, PCR can introduce artifacts that mimic true variants. To counter this, barcodes known as unique molecular identifiers (UMIs) are applied to original DNA templates before amplification so that all descendant copies inherit the same identifier, enabling discrimination of PCR-induced errors from true mutations [37]. Capture-based methods replace the initial error-prone PCR step with probe hybridization to enrich target DNA fragments, which are captured by streptavidin-coated beads for sequencing. Two widely studied hybrid-capture systems are Cancer Personalized Profiling by deep Sequencing (CAPP-Seq) and Targeted Error Correction Sequencing (TEC-Seq) [38]. Both use UMIs to suppress random sequencing errors but add further safeguards. CAPP-Seq couples UMI consensus with integrated Digital Error Suppression (iDES), a position-specific statistical filter that predicts and removes recurrent artifacts [39]. TEC-Seq adopts a rule-based confirmation scheme to suppress artifacts rather than position-specific error models [40]. Currently, FoundationOne Liquid CDx is the only FDA-approved cfDNA capture-based companion diagnostic for detection of *BRCA1/2* and *ATM* alterations in mCRPC [https://www.fda.gov/drugs/drug-approvals-and-databases/fda-approves-liquid-biopsy-next-generation-sequencing-companion-diagnostic-test (accessed on 28 April 2026)]. Other assays remain investigational. Using a CAPP-Seq-based workflow, Dang et al. identified alterations in the AR enhancer, the AR gene, and 84 other genes in patients with mCRPC. [41]. Patients with AR and enhancer alterations had worse outcomes (6-month PFS 30% vs. 71%, *p* = 0.0002; 6-month OS 59% vs. 100%, *p* = 0.0015). AR enhancer alterations alone were also associated with shorter PFS (*p* = 0.0001) and OS (*p* = 0.0004) [41]. Notably, liquid biopsy can also identify BRCA reversion mutations, which restore the gene’s open reading frame, leading to resistance to PARP inhibitors (PARPIs) [42]. In 2017, Quigley et al. profiled cfDNA from two mCRPC patients progressing on PARPIs using a capture-based assay and detected more BRCA2 reversion alleles than in single-site tissue biopsy [43]. More recently, a post-progression cfDNA analysis with the Guardant360 hybrid-capture assay in the TRITON2 study reported somatic BRCA reversions in 39% of cases, with higher reversion rates in patients under prolonged drug pressure (time on treatment 8.2 vs. 5.3 months, HR 0.43, 95% CI 0.27–0.60, *p* < 0.001) [44].

Untargeted detection approaches include whole-genome sequencing (WGS), which surveys the entire genome, and whole-exome sequencing (WES), which captures only coding regions (1–2% of the entire genome) at higher depth. In the TOPARP-A trial, Goodall et al. performed cfDNA whole-exome sequencing (WES) on six samples obtained at disease progression, identifying newly acquired aberrations in *BRCA2*, *PALB2*, *ARID1A*, *TP53*, and *TSC2* [38,45]. Notably, untargeted methods are most informative when the DNA tumor fraction (TFx) is ≥10%, which occurs more frequently in advanced prostate cancer [46]. To improve diagnostic performance at lower TFx, WGS-based prescreening systems quantifying genome-wide copy number alterations (CNAs) have emerged [47]. In this regard, ichorCNA is a computational tool developed at the Broad Institute of the Massachusetts Institute of Technology (MIT) and Harvard University that estimates TFx from ultra-low pass WGS to select cfDNA samples with sufficient tumor content for untargeted analyses. In mCRPC patients, Francini et al. used ichorCNA to preselect adequate-TFx samples and evaluate the association between ABCB1 amplification and primary resistance to Docetaxel or Cabazitaxel chemotherapy [48]. Another method for TFx estimation is modified Fast Aneuploidy Screening Test-Sequencing (mFAST-SeqS). Researchers have used this amplicon-based NGS assay to stratify mCRPC ARPI-treated patients using a genome-wide aneuploidy (GWA) score. Higher GWA scores correlated with higher tumor fractions (*p* < 0.001) and independently predicted worse overall survival (score ≥ 5 vs. <5: HR 2.49; 95% CI 1.57–3.97; *p* < 0.001) [49].

Beyond genetic biomarkers, third-generation “analog” strategies interrogate epigenetic features such as ctDNA methylation and fragmentation patterns. Unlike the former “digital” approaches that analyze predefined gene variants at discrete loci, these methods capture subtle, continuous shifts across thousands of sites [27,28]. Methylomics analyzes the pattern of methylations in the genome. In cancer, global hypomethylation combined with focal promoter hypermethylation of tumor-suppressor genes has been described [50,51]. cfDNA methylomic investigations in prostate cancer explored the predictive and prognostic potential of epigenetic alterations of genes such as *GSTP1*, *APC*, *AR*, *RASSF1A*, *PTGS2*, and *MDR1* [52]. In mCRPC, a post hoc analysis of the phase 3 SYNERGY trial showed that detection of a methylated *GSTP1* (mGSTP1) gene in cfDNA holds an independent prognostic value. A longer OS was observed in patients with undetectable baseline mGSTP1 and those achieving mGSTP1 plasma clearance after two Docetaxel cycles (HR 0.40, 95% CI 0.29–0.55, *p* < 0.00001 and HR 0.36, 95% CI 0.29–0.46, *p* < 0.00001, respectively) [53]. In another study with mCRPC patients treated with Abiraterone or Enzalutamide, the presence of an *AR* gene hypomethylation signature (AR-MethSig) was associated with *AR* copy number gain (*p* < 0.001) and significantly shorter OS compared with the absence of AR-MethSig (HR 8.18; *p* = 0.0044) [54]. Wong et al. developed a risk stratification model combining methylomic profiling with clinical biomarkers such as PSA, LDH, and ALP: high-risk patients had a significantly shorter PFS than low-risk patients (14.4 vs. 42.8 months; *p* = 0.00014) [55]. Notably, to improve diagnostic accuracy, researchers developed a cfDNA-based method to detect neuroendocrine differentiation in mCRPC with high accuracy (AUC > 0.93), identifying aggressive tumors associated with ARPI resistance [56]. Additionally, recent studies have reported cfDNA methylation assays using target enrichment and machine learning to improve early metastasis detection. Chen et al. identified metastatic cases with high accuracy (AUC = 0.989) and observed widespread hypermethylation with pericentromeric hypomethylation, suggesting genomic instability [57]. Finally, methylomics applications can effectively address ultra-low genomic coverage settings, such as early cancer detection where ctDNA shedding is minimal, and emerging technologies such as Oxford Nanopore sequencing have been shown to enable accurate tissue-of-origin identification [58].

Another emerging strategy is fragmentomics, the analysis of cfDNA fragment size and structure. ctDNA fragments correspond to mono- and di-nucleosomal units generated by nuclease cleavage [59,60] and are typically shorter than non-tumor cfDNA, ranging from ~60 to 145 bp [61]. In patients with mCRPC, cfDNA fragmentation analysis demonstrated:

i. Significantly shorter mean fragment lengths compared with localized disease (*p* < 0.05);
ii. An inverse correlation between fragment size and circulating tumor fraction (*p* < 0.0001);
iii. A shorter PFS and OS in patients with higher proportions of longer fragments (*p* = 0.008 and *p* = 0.027, respectively) [57]. Notably, integrating fragment size profiles with cfDNA end-motif patterns enables the reconstruction of nucleosome footprints (i.e., “nucleosomics”) that reflect chromatin accessibility and transcription factor binding, thus informing gene expression and tissue of origin [28]. De Sarkar et al. applied patient-derived xenograft (PDX) nucleosome-footprint signatures to classify human mCRPC phenotypes. In 101 cfDNA samples from the Dana-Farber Cancer Institute database, the model distinguished adenocarcinoma from neuroendocrine subtypes with an overall accuracy of 0.96 (AUC 0.97 for high tumor fraction, TFx ≥ 0.10; 0.76 for low tumor fraction, TFx < 0.10) [62].

In general, the choice of detection method should be aligned with the clinical setting, as ctDNA levels vary according to tumor burden, sites of disease, and treatment response [21,63,64,65]. In advanced prostate cancer, where cfDNA fractions in plasma are likely higher, digital targeted and untargeted approaches may be the most appropriate option. Conversely, in earlier states of disease, typically associated with low tumor DNA shedding levels, the emerging epigenomic assays may offer broader detection capabilities; however, large prospective studies are required to further validate these novel technologies.

## 3. Role of ctDNA in Localized Prostate Cancer

Currently, there is no established clinical role for ctDNA in localized prostate cancer, including high-risk cases, due to the exceedingly low ctDNA shedding, thus reducing the sensitivity of second-generation liquid biopsy assays [66].

In a cohort of 112 patients with localized prostate cancer, investigators used ultra-low-pass whole-genome and targeted sequencing to analyze patient-specific plasma mutations, reporting no detectable ctDNA [67]. In contrast, Zang PD et al. utilized a novel tumor-informed ctDNA assay, integrating enriched amplicons and UMIs [68]. ctDNA was detectable in 5 out of 11 patients preoperatively and in 7 out of 11 following surgery, with postoperative ctDNA detection being associated with disease relapse [68].

Furthermore, Lau E et al. studied WGS of tumor–normal pairs in eight patients diagnosed with localized disease who were undergoing prostatectomy [69]. Tumor variants in ctDNA were observed in 2 out of 8 patients and remained detectable postoperatively, correlating with rapid disease recurrence and progression. In a separate cohort of 189 patients, the detection of TP53 in ctDNA (12%) was linked to a shorter metastasis-free survival [69].

In another study, ctDNA detection in 19 (9.3%) out of 118 localized prostate cancer patients at the time of prostatectomy was associated with significantly shorter biochemical recurrence-free survival and metastasis-free survival compared with patients with undetectable ctDNA [65]. Conversely, a high accuracy was assessed by Chen S. et al. (AUC = 0.989) using methylated cfDNA immunoprecipitation coupled with NGS (cfMeDIP-seq) in correctly classifying all localized samples (n = 60) and misclassifying only 10 of 175 metastatic samples; notably, nearly all cases with TFx < 2% were correctly classified [57].

In conclusion, despite the promising findings from ctDNA epigenomic profiling approaches in localized prostate cancer, imaging modalities such as multiparametric MRI and PSMA-PET together with PSA testing currently remain the gold standard for diagnosis and disease monitoring.

## 4. Role of ctDNA in Advanced Prostate Cancer and Its Clinical Application

The widespread adoption of NGS has greatly enhanced our understanding of the complexity of the heterogeneous genomic landscape of advanced prostate cancer. Commonly mutated genes identified in primary prostate cancer include *SPOP*, *FOXA1*, *TP53*, and *PTEN* [70]. A molecular study conducted by the Cancer Genome Atlas Research Network on 333 primary prostate tumors revealed that approximately 53% of the tumor samples exhibited ETS gene fusions, 11% included *SPOP* mutations, 15% showed homozygous deletions of *PTEN*, and 19% had alterations in DNA damage response (DDR) genes [71]. Among 424 patients with mHSPC assessed by Stopsack et al., commonly altered genes included *TP53* (33%) and *PTEN* (24%), followed by *FOXA1* (13%) and *SPOP* (13%) [72]. Notably, patients with high-volume disease frequently harbored mutation of *NOTCH*, a gene involved in the cell cycle, as well as in genes associated with epigenetic modifiers. Moreover, de novo metastatic disease exhibited a higher prevalence of CDK12 alterations compared with patients with metachronous disease [72].

Conversely, in patients with mCRPC a greater frequency of alterations in AR signaling pathways (71%) has been observed [73], together with 49% of the cases displaying alterations in the PI3K pathway, 41% *PTEN* gene mutations, 57% ETS gene mutations, 53% *TP5*3 gene mutations, and 23% DDR gene mutations [73]. Importantly some of these alterations are currently actionable, including PTEN loss [74,75,76] and HRR and BRCA mutations [77], and/or hold a prognostic potential (i.e., PTEN deficiency, SPOP mutations [78], HRR and BRCA mutations, [79], etc.) [80]. 

Furthermore, the timing of sample collection plays a crucial role, as ctDNA levels fluctuate according to the clinical setting [81]. In a single-institution study involving 100 patients (77 with mCRPC and 23 with mHSPC), the probability of ctDNA positivity was higher when sampling occurred during radiographic or clinical progression (*p* = 0.0022 and *p* = 0.0111, respectively), whereas no correlation with PSA progression was observed (*p* = 0.1996) [82]. Additionally, a large prospective study conducted in Japan reported a higher prevalence of alterations in patients with mCRPC compared to those with mHSPC, involving AR (28.4% vs. 0%, *p* < 0.0001), BRCA2 (15.8% vs. 4.4%, *p* = 0.024), ATM (15.8% vs. 1.5%, *p* = 0.0023), and HRR defects (42.1% vs. 20.6%, *p* = 0.0043) [83]. Similarly, in a longitudinal study by Kohli et al., ctDNA results differed significantly between mHSPC and mCRPC settings (*p* < 0.001), with significantly increased AR and APC mutations in the mCRPC state compared with mHSPC (*p* < 0.05). Notably, a high baseline ctDNA fraction in mHSPC was predictive of a significantly shorter time to ADT failure (*p* = 0.02), highlighting the importance of early testing [84]. 

According to recent American Society of Clinical Oncology (ASCO) guidelines, while archival tissue remains the preferred initial approach for somatic testing, ctDNA is recommended when metastatic sites are inaccessible for biopsy or when sequential testing is required [85]. To date, the only FDA-approved companion diagnostic in mCRPC is the FoundationOne Liquid CDx assay, used to detect BRCA and ATM alterations [https://www.fda.gov/drugs/drug-approvals-and-databases/fda-approves-liquid-biopsy-next-generation-sequencing-companion-diagnostic-test (accessed on 28 April 2026)].

Supporting evidence derives from the phase 3 PROfound study, which evaluated Olaparib versus ARPIs in ARPI-pre-treated patients with mCRPC harboring HRR gene alterations [86]. Of the >4000 patients screened for this study, approximately one-third did not yield an interpretable tissue NGS result. Specifically, 11.7% of samples failed the initial pathology review due to inadequate tumor content, and 22.7% of the remaining samples failed during DNA extraction because of low DNA yield. Additional failures were due to poor nucleic acid quality in decalcified bone metastases and older archival samples, with NGS success rates decreasing to 47.3% for specimens older than 10 years. To overcome these constraints in tissue availability, the trial prospectively incorporated the FoundationOne Liquid CDx ctDNA assay, which demonstrated 81% concordance with matched tissue for detecting BRCA and ATM alterations, ultimately supporting its regulatory approval [86].

## 5. ctDNA as Prognostic Biomarker

Evidence from several clinical trials highlights the prognostic impact of baseline cfDNA levels in mCRPC. In the phase 2 TheraP trial, patients with ctDNA fractions < 2% had a longer OS than those with 2–30% fractions (median, 34 vs. 19, HR 3.0, *p* < 0.001) and those with >30% fractions (median, 34 vs. 9.9, *p* < 0.0001) [87]. Consistent findings were observed in the phase 3 TALAPRO-2 trial, where a high ctDNA burden at baseline was associated with shorter rPFS in both treatment arms [88]. Notably, patients who converted from high to low ctDNA within the first 9 weeks of treatment had longer rPFS, while those who remained ctDNA-low throughout therapy showed the most favorable outcomes [88]. In the phase 3 PSMAfore trial, dynamic changes in ctDNA were also clinically informative: fractional decreases in ctDNA between baseline and cycle 2 day 1 were significantly associated with a longer rPFS (*p* = 0.0015) and correlated more strongly with a longer OS than PSA declines (*p* = 0.002 vs. *p* = 0.12, respectively) [89]. Finally, a recent systematic review and meta-analysis indicated that higher baseline ctDNA levels were associated with shorter PFS and OS in prostate cancer patients across 13 studies, confirming its potential role as a prognostic biomarker [90].

## 6. ctDNA in Metastatic Hormone-Sensitive Prostate Cancer

In a study on 53 patients with de novo mHSPC, the median ctDNA fraction was 11% (0–84%) [63]. ctDNA levels were higher in cases with visceral metastases compared to those with bony or lymph node-only disease (8/8 vs. 14/26 patients, respectively; *p* = 0.03). The study identified plasma TP53 mutations in 47% of patients and DDR gene alterations in 21%. Although ctDNA and prostate biopsy showed 80% concordance, either modality alone failed to detect clinically relevant somatic alterations in 36% of cases, highlighting their complementary role. A key limitation of this study was the use of a narrow gene panel for sequencing. Similarly, a larger retrospective study of 182 HSPC patients showed that ctDNA positivity increased with disease burden, reaching 54.2% in high-volume mHSPC compared with 14.3% in low-volume and 8.3% in localized disease. The presence of pathogenic alterations in BRCA1/2, TP53, PTEN, RB1, or CDK12 in either tissue or ctDNA was associated with a shorter time to CRPC (median, 12.2 months vs. NR; HR 2.97, 95% CI 1.35–6.52; *p* < 0.005); this association remained highly significant in a multivariable analysis (HR 3.3; 95% CI 1.5–7.3; *p* = 0.004) [91]. This supports the prognostic role of these alterations in mHSPC, consistent with large tissue-based analyses [79,92].

The SCRUM-Japan MONSTAR SCREEN project evaluated ctDNA in both mHSPC and mCRPC patients, showing that HRR gene alterations were associated with a significantly shorter time to CRPC in patients treated with ADT or ADT plus first-generation antiandrogens (HR 6.12, 95% CI 1.80–20.8; *p* = 0.0037), but not with ADT plus ARPIs (HR 0.37, 95% CI 0.043–3.20; *p* = 0.37) [83]. These results are consistent with findings from RNA profiling studies of tumor samples [79]. Additionally, in the mCRPC setting, AR amplification and AR mutations were associated with a shorter time to treatment failure with ARPIs (*p* < 0.0001 and *p* = 0.012, respectively).

In a cohort of 66 patients with mHSPC receiving chemotherapy combined with hormonal therapy, Xu et al. demonstrated that early ctDNA dynamics predict treatment efficacy [93]. After one cycle, patients without ctDNA increase (65.2%) had a longer time to CRPC than those with increased ctDNA (34.8%) (17.70 vs. 8.43 months; *p* < 0.001). Early ctDNA increase was an independent predictor of shorter time to CRPC (HR 2.28, 95% CI 1.22–4.25; *p* = 0.010). Furthermore, the emergence of post-treatment HRR alterations was associated with a faster progression to CRPC (8.02 vs. 13.20 months; *p* = 0.011) compared with their absence. Table 1 summarizes the key findings from some of the studies mentioned above.

## 7. ctDNA in Metastatic Castration-Resistant Prostate Cancer

Considering the heterogeneity of mCRPC, genomic re-characterization of the disease at this advanced stage holds the potential to unveil newly actionable gene alterations. However, achieving new tissue biopsies is an invasive procedure, and DNA extraction is particularly challenging in the case of bone metastases—the most common type of metastases in mCRPC—due to decalcification-related DNA damage [106]. In contrast, ctDNA analysis represents a promising non-invasive option for molecular profiling. In this regard Fonseca et al. [107] investigated 738 plasma cfDNA samples obtained from 491 patients with mCRPC across two phase II randomized clinical trials and a prospective province-wide blood biobanking program [21]. Their findings indicated that patients with a ctDNA fractions > 30% had the shortest OS (11.1 months vs. 15.7 months for 2–30% and 25.0 months for <2%) and a five-fold increased risk of PSA progression during first-line treatment (*p* < 0.001) and a 5.6-fold higher risk of mortality (*p* < 0.001) compared with those with a ctDNA fraction < 2%. In addition, baseline ctDNA levels correlated with metastatic disease distribution, with higher fractions observed in liver metastases (median 42%, detectable in 90% of patients) compared with lymph node-only metastases (median 4.9%, detectable in 57% of cases; *p* < 0.001). In bone-only disease, ctDNA levels increased with tumor burden, with a median fraction of 10.4% in patients with ≥10 bone lesions compared with 3.9% in those with fewer lesions (*p* < 0.001). Intriguingly, by integrating cfDNA with routinely collected clinical parameters such PSA, alkaline phosphatase, LDH, hemoglobin, albumin, and metastatic disease distribution, the authors developed a machine learning model capable of accurately predicting the presence of a suitable ctDNA fraction (≥2%) for genomic profiling with high predictive accuracy (AUC = 0.76–0.80).

Similarly, data from a phase II randomized clinical trial in patients with poor prognosis mCRPC, comparing Cabazitaxel with Abiraterone or Enzalutamide, revealed that higher than median baseline ctDNA fractions (≥15%) were associated with shorter time to progression (HR 2.38; *p* < 0.001) and reduced OS (HR 3.71; *p* < 0.001) compared with <15%. The correlation was further confirmed by Choudhury et al., who demonstrated TFx strongly associates with the number of bone metastases (median TFx 1.4% for 0 vs. 19.0% for ≥4 lesions; *p* < 0.0001) and the presence of visceral metastases (median TFx 34.0% vs. 7.7% for bone-only disease; *p* < 0.0001). Notably, any new treatment yielding a ≥30% PSA decline at 6 weeks resulted in a TFx decrease (median change −92.2%) in all patients with a baseline TFx > 7% [108].

In the ProBio platform trial, of 220 patients with mCRPC [95], 139 had detectable ctDNA. Patients with a detectable ctDNA fraction (≥2.5%) experienced a 51% shorter OS compared with those with undetectable ctDNA (median, 22.2 vs. 45.6 mo.; survival-time ratio, 0.49; 90% CrI, 0.38–0.61). The dose–response analysis showed a clear linear relationship: for every 10-point increment in the ctDNA fraction, a patient’s expected survival and clinical benefit times were reduced by an additional 10% [95].

Longitudinal monitoring of ctDNA tumor fraction from the IMbassador250 phase III clinical trial data provided valuable insights in patients treated with Enzalutamide after Abiraterone [96]. Baseline ctDNA detection was associated with significantly shorter median OS (13.6 vs. 22.5 months; *p* < 0.001) and rPFS (4.6 vs. 11.4 months; *p* < 0.001) compared with patients without detectable ctDNA. ctDNA persistence at cycle 3 day 1 (C3D1) was associated with poorer outcomes compared with ctDNA clearance (OS 12.6 vs. 22.1 months; *p* < 0.001; rPFS 4.6 vs. 15.0 months; *p* < 0.001). Importantly, patients with undetectable ctDNA but no PSA reduction had better outcomes than those with detectable ctDNA despite a PSA response (median OS 22.1 vs. 16.0 months; *p* < 0.001).

Furthermore, 571 plasma samples were prospectively collected from large phase III clinical trials (FIRSTANA, PROSELICA) in patients receiving first-line (Docetaxel or Cabazitaxel) and second-line chemotherapy (Cabazitaxel) [109]. Higher baseline cfDNA levels were independently associated with shorter rPFS (HR 1.54, 95% CI 1.15–2.08; *p* = 0.004) and OS (HR 1.53, 95% CI 1.18–1.97; *p* = 0.001). Additionally, a decline in cfDNA concentrations during the first four cycles of systemic treatment was significantly associated with a PSA decrease (*p* = 0.003).

ctDNA analysis can help predict resistance to ARPI in mCRPC patients and can guide treatment decisions. In a study involving 202 treatment-naïve patients with mCRPC, participants were randomized to receive either Abiraterone or Enzalutamide [98]. The identification of genomic alterations in genes such as *BRCA2*, *ATM*, *TP53*, and *AR* rearrangements was associated with poor clinical outcomes and resistance to ARPIs. Furthermore, In a study involving 81 treatment-naïve patients with mCRPC treated with Abiraterone or Enzalutamide, persistent ctDNA detection at 4 weeks was an independent strong predictor of shorter PFS (HR 4.98; 95% CI 2.08–11.93, *p* < 0.001) and OS (HR 3.69; 95% CI 1.50–9.08, *p* = 0.005) [110]. A phase II trial involving 151 mCRPC patients treated with Abiraterone found that pre-treatment or cycle 2 *TP53*, *RB1*, or *PTEN* alteration detection correlated with shorter OS, compared with undetectable ctDNA cases [111].

In the phase 2 TheraP trial the role of ctDNA was investigated in 178 patients randomized to Lutetium-177-PSMA-617 (Lu-PSMA-617) or Cabazitaxel [87]. A low or undetectable baseline ctDNA fraction predicted higher biochemical response rates (100% vs. 58%, *p* = 0.0067) and longer mPFS (14.7 vs. 6.0 months, *p* = 0.00025) in the Lu-PSMA-617 arm compared with Cabazitaxel, independent of PSMA PET biomarkers, although no significant OS benefit was observed. Additionally, patients with *PTEN* loss derived a significant OS benefit from Lu-PSMA-617 compared with Cabazitaxel (HR 0.39; *p* = 0.022) [87]. Importantly, AR gene alterations showed no influence over clinical outcomes, except in a subgroup of patients harboring ≥ 16 AR amplifications, who exhibited a shorter OS with 177Lu-PSMA-617 compared with Cabazitaxel (*p* = 0.025) in an univariable analysis [87]. In contrast a phase 2 study of Lu-PSMA-617 in heavily pretreated mCRPC patients (*n* = 40) found that patients with baseline ctDNA-detected *AR* amplification were associated with a shorter mPFS (4.7 vs. 9.4 months; *p* = 0.020) and mOS (7.4 vs. 19.1 months; *p* = 0.020) compared with those harboring a normal *AR* gene [112].

In a prospective Lu-PSMA-617 registry (*n* = 150), Fettke H et al. reported that undetectable baseline ctDNA was independently associated with a longer PSA-PFS (HR 0.50, 95% CI 0.29–0.86; *p* = 0.017), a longer OS (HR 0.45, 95% CI 0.25–0.83; *p* = 0.019), and higher PSA50 response rates (83% vs. 51%; *p* = 0.002) [99]. Notably, the persistence of ctDNA negativity at week 6 independently predicted longer PFS and OS (*p* < 0.001 and *p* = 0.002, respectively). ctDNA clearance at week 6 was associated with favorable outcomes (median PSA-PFS 7.2 months; mOS 18.6 months). In contrast, persistent ctDNA detection independently predicted shorter outcomes (PFS HR 2.45, 95% CI 1.31–4.42; *p* = 0.004; OS HR 2.90, 95% CI 1.41–5.80; *p* = 0.003). Additionally, deleterious *FOLH1* gene alterations were associated with a shorter PSA-PFS with 177Lu-PSMA-617 (HR 3.67, 95% CI 1.33–10.0; *p* = 0.011) [99]. 

A recent study used cfDNA to analyze epigenomic signals from promoters, enhancers, and DNA methylation in patients with mCRPC (*n* = 85) to evaluate resistance mechanisms against 177Lu-PSMA-617 therapy [113]. The increased expression of the *FOLH1* gene was independently associated with longer clinical and radiographic PFS (crPFS; *p* < 0.01) and OS (*p* < 0.01). In contrast, high AR enhancer gene activity was associated with worse crPFS (HR 1.89, *p* = 0.049). Immune-related signaling, including TNFR1 pathway activation, correlated with improved crPFS (HR 0.38, FDR 0.08), whereas WNT signaling correlated with resistance and shorter crPFS (HR 2.17, FDR 0.05). Interestingly, epigenomic signals (i.e., *CHGA*, *DLL3*, and *SEZ6* genes) consistent with neuroendocrine differentiation were identified in a small subset of patients, with trends toward shorter OS.

By analyzing cfDNA samples from 776 men with mCRPC undergoing fist-line ARPI treatment in the phase 3 trial Alliance trial, the emergence of *AR*, *MYC*, *RSPO2*, *ZBTB16*, *PTEN*, *CHD1*, *TP53*, and *RB1* gene alteration may predict prognosis [114]. The authors developed and validated a clinical genetic (CG) model for predicting OS by integrating ctDNA-detected pathogenic genomic alterations with standard clinical variables (e.g., hemoglobin, alkaline phosphatase, albumin, ECOG performance status, presence of visceral metastases, LDH, PSA levels, etc.). This model outperformed standard clinical criteria (time-dependent AUC 0.77 vs. 0.72; *p* = 0.01) enabling patient stratification into four risk groups: poor (median OS, mOS 17.3 months), intermediate–poor (mOS 31.1 months), intermediate–low (mOS 45.1 months), and low-risk (mOS 64.2 months).

Zhao et al. employed longitudinal ctDNA profiling to capture the heterogeneous nature of mCRPC under treatment pressure. In a multi-trial analysis of 60 patients a higher baseline ctDNA fraction (>13.7%) was associated with worse OS (*p* = 0.03) [100]. The authors also introduced the Evolutionary Dynamic Index (EDI) to assess shifts in subclonal frequency during treatment [100]. A low EDI was associated with shorter OS (*p* = 0.028), suggesting pre-existing resistant clones. Lower clonal heterogeneity (≤4 subclones) was associated with longer OS compared with tumors having >4 subclones (*p* = 0.032). ARPIs caused more significant evolutionary changes compared with Taxanes (*p* = 0.0422). ARPI treatment led to resistant subclones with AR amplifications and alterations in PI3K-AKT and DNA damage response genes, while treatment with Taxane was associated with changes in epigenetic regulators and structural proteins. Table 1 and Table 2 highlight key findings from several studies that utilized ctDNA.

## 8. ctDNA as Predictive Biomarker

An increasing body of evidence supports the role of cfDNA in the identification of actionable mutations for personalized medicine approaches in mCRPC. This section focuses on ctDNA-based biomarkers relevant for treatment selection.

HRR gene alterations are commonly found in 20–30% cases of mCRPC [22,73,126,127,128], more commonly *BRCA1/2* and *ATM* genes, while *CHEK2*, *PALB2*, and *CDK12* gene mutations occur less frequently. In a large cohort of 3334 mCRPC patients, ctDNA analysis detected *BRCA1/2* alterations in 8.8% of cases [129]. The study reported a high concordance rate of 93% between tissue and liquid biopsy results, with 100% detection of germline variants in ctDNA. Notably, BRCA1/2 alterations were identified exclusively in ctDNA in 2.4% of cases, including somatic, subclonal, and reversion mutations, supporting the complementary role of ctDNA in diagnosis and treatment monitoring.

Currently, several PARPIs such as Olaparib, Talazoparib, Rucaparib, and Niraparib have been approved by the FDA for use either as monotherapy or in combination with ARPIs [130,131,132,133]. Particularly, the three landmark phase III randomized clinical trials PROpel, TALAPRO-2, and MAGNITUDE supported the approval of the addition of a PARPI to an ARPI as first-line in mCRPC [134,135,136]. Of note, these trials included the use of ctDNA-based liquid biopsy as a screening tool for genomic profiling. This approach was also adopted in the AMPLITUDE trial, which recently led to the FDA approval of Niraparib plus Abiraterone and prednisone for patients with mHSPC and BRCA 1/2 aberrations [137].

Microsatellite instability-high (MSI-H) or mismatch repair deficiency (dMMR) is found in approximately 2–3% of advanced prostate cancer cases, based on studies that utilized tissue NGS [73,138,139]. Pembrolizumab is approved for advanced tumors including mCRPC with tumor-agnostic criteria in patients with MSI-H status or high tumor mutational burden (TMB), defined as ≥10 mutations per megabase, who progressed on available standard treatments [140,141,142]. Several case reports have demonstrated excellent clinical response to pembrolizumab in patients with mCRPC harboring MSI-H detected through liquid biopsy [118,119,143]. To date, ctDNA-based MSI-H testing is not FDA-approved, and ctDNA-based high tumor mutational burden (TMB) testing remains investigational [101,144]. However, the European Society for Medical Oncology (ESMO) guidelines recommend ctDNA testing for MSI-H when tumor tissue is unavailable [145].

Alterations in the PTEN-PI3K-AKT pathway are frequently found in liquid biopsies of mCRPC patients and are associated with aggressive disease and treatment resistance [98,105,115,116]. In the phase 3 trial IPATEntial150 of patients with previously untreated mCRPC and PTEN loss identified through immunohistochemistry (IHC), the combination of the AKT inhibitor ipatasertib and Abiraterone/prednisone significantly improved rPFS [75,76]. The most notable benefits were seen in specific genomic subgroups, particularly those with alterations in the PI3K/AKT pathway. In patients with de novo metastatic hormone-sensitive prostate cancer exhibiting PTEN loss via immunohistochemistry, the CAPItello-281 phase III Trial showed that the AKT inhibitor Capivasertib combined with Abiraterone/prednisone plus ADT led to a significant improvement in rPFS compared to placebo plus Abiraterone/prednisone and ADT alone; final overall survival data are pending [74]. These findings support a biomarker-driven treatment approach in advanced prostate cancer. In this regard, ctDNA may facilitate patient selection in future trials by identifying actionable alterations such as PTEN loss and *PIK3CA* mutations.

Finally, as highlighted in other sections, ctDNA dynamics represent a key predictive feature. In the phase III TALAPRO-2 trial, patients whose ctDNA burden converted from high to low by week 9 had longer median rPFS than those remaining ctDNA-high, regardless of the treatment arm. However, outcomes remained more favorable in patients with persistently undetectable ctDNA compared with those converting from high to low [88].

Additional selected examples are summarized in Table 2, along with ctDNA-based findings on key predictive genomic alterations with therapeutic implications.

## 9. Using Liquid Biopsies to Predict Resistance to Systemic Treatments for Advanced Prostate Cancer

Longitudinal monitoring with ctDNA comprehensive genomic profiling is a promising technique for identifying key resistance mechanisms that contribute to disease progression and neuroendocrine transformation during systemic treatments.

Alterations in AR pathways represent a common mechanism of resistance to systemic treatments using ARPI and for the progression to mCRPC [102,104,146,147,148,149,150]. The presence of AR amplification in cfDNA is associated with a limited response to ARPI, resulting in poor PFS and OS in mCRPC, indicating a more aggressive disease [123]. Crucially, upfront intensified treatment with Abiraterone and/or Enzalutamide may exert strong selective pressures for genomic changes in AR pathways [151]. Through longitudinal cfDNA profiling in first-line mCRPC patients treated with Enzalutamide, with or without Abiraterone, Valentín López JC et al. revealed distinct resistance patterns based on AR dependence [125]. Of 1311 participants, 327 had matched baseline and progression plasma samples suitable for analysis. Rapid progressors (rPFS ≤ 6 months) exhibited more non-AR alterations, including *TP53*, *PTEN*, *RB1*, *FANCA*, *ETS2*, and *ZNRF3* (at baseline and at progression), consistent with AR-independent or lineage-plastic mechanisms [125]. In contrast, delayed progressors (rPFS ≥ 30 months) showed a higher prevalence of AR alterations, including copy number gains and genomic rearrangements, with fewer non-AR events.

Mutations in the AR ligand-binding domain (LBD) or rearrangements of the AR gene are common, occurring in 10–40% of metastatic mCRPC cases, as identified through liquid biopsies in the published literature [102,103,129,149]. ctDNA-based detection of AR LBD is essential for understanding ARPI resistance mechanisms. Hotspot mutations in the LBD of the androgen receptor, such as *H875Y* and *T878A*, lead to a more promiscuous AR protein that can be activated by various endogenous steroids, including glucocorticoids, estrogen, and progesterone [122,146,147,152,153]. The *AR L702H* mutation is frequently observed following treatment with Enzalutamide and Abiraterone and is associated with poorer OS [122]. Importantly, *L702H* is specifically activated by exogenous steroids, which is noteworthy because patients receiving prednisone with Abiraterone or Docetaxel may activate these mutations, resulting in treatment resistance [154].

The emergence of AR LBD alterations, including *W742C/L*, *H875Y*, and *T878A*, is known to confer agonist activity to first-generation ARPIs such as bicalutamide and flutamide [148,153,155,156]. Moreover, *F877L* and *T878A* mutations can exhibit partial agonist activity with Enzalutamide and Apalutamide [157,158,159]. Other evidence indicates the emergence of AR *T878A/S*, *AR H875Y*, *F876L*, *H874Y*, *T877A* and *L702H* mutations after treatment with Enzalutamide and Abiraterone, correlating with lower PSA responses [103,120,121,124]. Patients harboring multiple aberrant AR mutations (two or more LBD alterations) tend to experience significantly worse clinical outcomes and rapid progression of disease [103,122]. In a phase II ctDNA substudy of the Canadian Cancer Trials Group (CCTG), Darolutamide showed modest clinical activity in patients with mCRPC who had previously been treated with ARPIs such as Abiraterone, Enzalutamide, and Apalutamide [160]. Clinical benefit was observed in patients with *SPOP* mutations, AR amplifications or *L702H* and *T878A* [160]. Conversely, the presence of *AR F877L*, *W742C/L*, and *V716M* was not associated with significant benefit.

RNA AR splice variants determine structural rearrangements to the AR protein, which lacks a ligand-binding domain while retaining active transcriptional N-terminal activity [161]. The most prevalent form is the AR splice variant 7 (AR-V7), which is associated with resistance to newer ARPIs (Apalutamide, Enzalutamide, and Abiraterone) and worse clinical outcomes [162,163]. Currently, ctDNA-based assays available in clinical practice cannot detect AR-V7. However, AR-V7 can be identified in liquid biopsies through RNA-based methods that analyze circulating tumor cells and exosomal RNA [162,164].

PARP inhibitors have demonstrated improved clinical outcomes in patients with advanced prostate cancer; however, they are often associated with the development of resistance to the treatment, particularly through *BRCA* reversion mutations. NGS using ctDNA assays is an effective method for detecting these *BRCA* reversion mutations. While NGS-based ctDNA analysis is recommended per NCCN guidelines when tissue samples are unavailable in mCRPC for identifying actionable alterations, including those in HRR genes, there are currently no specific recommendations for the serial use of ctDNA to monitor emerging reversion mutations during therapy [7]. The first reported case of *BRCA2* reversion mutations was identified in a patient with mCRPC treated with carboplatin, harboring a germline *BRCA2* frameshift mutation (p.N2452Mfs*17) [165]. Initially, the patient achieved > 90% PSA reduction. However, upon progression, ctDNA-targeted NGS analysis revealed the emergence of 90 BRCA2 mutations, 17 of which (19%) exhibited secondary indel variants restoring the gene’s reading frame, suggesting polyclonal reversion as a mechanism of resistance to platinum therapy. The pivotal trial TRITON2 [166] evaluated ctDNA for *BRCA* reversion mutations in patients with mCRPC who progressed on Rucaparib [44]. A total of 100 patients underwent ctDNA testing, and no baseline *BRCA* alterations were observed in those with known *BRCA1/2* mutations. However, at disease progression on Rucaparib, ctDNA analysis revealed BRCA reversion mutations in 39 patients, of whom 29 harbored ≥2 distinct reversion events, with similar rates observed between germline and somatic BRCA1/2 alterations. This study found that reversion mutations increased with the duration of Rucaparib treatment, suggesting that there is selective pressure for resistance in tumors that depend on BRCA loss for sensitivity to PARP inhibitors.

In conclusion, the above findings support and provide compelling clinical evidence for longitudinal monitoring of ctDNA to detect the emergence of resistance mutations, such as BRCA reversion and AR alterations, during treatment.

Table 2 outlines important genomic alterations related to predictive and resistance factors, which have significant clinical implications.

## 10. NEPC Marker Detection with Liquid Biopsies

Approximately 15–20% of patients with metastatic CRPC develop an aggressive phenotype of disease known as neuroendocrine prostate cancer (NEPC) [167,168,169]. This development signifies a resistance mechanism to ADT and ARPIs, driven by emergent lineage plasticity [169,170]. NEPC is associated with poor prognosis and exhibits an aggressive phenotype, with tumors that are independent of AR signaling. Currently, diagnosing NEPC poses challenges, as it necessitates invasive biopsies of the metastatic site for confirmation of aggressive phenotypes. However, liquid biopsy may overcome these limitations by offering a non-invasive approach to infer the NEPC phenotype through the analysis of ctDNA epigenetic patterns. Berchuck JE et al. evaluated tumor samples from patient-derived xenografts of NEPC and prostate adenocarcinoma using cell-free methylated DNA immunoprecipitation sequencing (MeDIP-seq) to identify differentially methylated regions (DMRs) specific to the NEPC subtype [171]. Based on these regions, the authors developed a NEPC risk score, which was subsequently tested in cfDNA samples, accurately distinguishing NEPC from CRPC adenocarcinoma (AUC 0.96, 100% sensitivity, 90% specificity). These findings were confirmed in an independent, multi-institutional validation cohort of 53 cfDNA samples (AUC 1.0, 100% sensitivity, 95% specificity). Notably, higher NEPC risk scores were significantly associated with shorter overall survival (OS) (HR 2.5, *p* = 0.017 in the test cohort; HR 4.3, *p* < 0.001 in the validation cohort) [171]. Franceschini GM et al. developed a non-invasive targeted plasma cfDNA methylation assay, termed neuroendocrine detection and monitoring (NEMO), to detect methylation patterns in CRPC with neuroendocrine features (CRPC-NE) [56]. This targeted assay quantified the overall tumor content in plasma and distinguished the CRPC adenocarcinoma subtype from the neuroendocrine one with an AUC of 0.93, which increased to an AUC of 0.97 in samples with high tumor fractions (>50%) [56]. Other investigators utilized a fragmentomic approach, analyzing ctDNA nucleosome positioning and fragment size patterns to differentiate AR-dependent adenocarcinoma from NEPC [62]. By evaluating fragmentation profiles corresponding to nucleosome positioning and cleavage patterns, they were able to reconstruct chromatin accessibility at specific transcription factor binding sites. This approach enabled the identification of distinct transcriptional regulatory programs of the two phenotypes directly from cfDNA WGS data, without the need for tissue RNA, achieving accuracy in estimating phenotype fractions within mixed clinical cases [62]. 

On the other hand, Zhao et al. used a clinical-grade multiplex RNA qPCR assay on CTCs, identifying NEPC through the expression of neuroendocrine markers, such as synaptophysin (SYP) and chromogranin A (CHGA), and the absence of AR target genes (e.g., *KLK2*, *KLK3*, *FOLH1*, and *TMPRSS2*) [172,173,174]. In an institutional cohort study involving 17 patients (7 with NEPC, 10 with adenocarcinoma), serial blood samples were collected, resulting in a total of 116 longitudinal CTC samples [174]. NEPC was indicated by the absence of AR target gene expression and the positive detection of neuroendocrine markers. While per-sample diagnostic sensitivity was moderate (51.3%), longitudinal serial monitoring achieved 100% accuracy in per-patient predictions. Notably, detecting neuroendocrine markers while retaining AR target expression significantly correlated with worse OS (*p* = 0.017) and a shorter time to treatment failure on ARSIs (*p* = 0.033) [172]. Consistently, other investigators evaluated the NETest, a 51-gene blood-based PCR transcriptomic assay capable of non-invasively detecting CRPC-NE by measuring the expression of plasma neuroendocrine neoplasia genes. NETest demonstrated superior diagnostic performance compared with standard PSA testing (AUC 0.93 vs. 0.70), yielding 94% sensitivity and 87% specificity [175].

Findings from the above studies emphasize the promising potential of enhanced diagnostic precision with non-invasive methods, utilizing ctDNA or CTCs, to detect aggressive phenotypes of CRPC. This approach paves the way for advancements in precision medicine for advanced prostate cancer.

## 11. ctDNA and Its Emerging Integration with Epigenomic Profiling

ctDNA assays mainly focus on detecting genetic alterations; however, epigenomic profiling provides a more comprehensive approach to tumor characterization and monitoring. This approach captures multiple layers of gene regulation, including histone modifications at promoters and enhancers, as well as patterns of DNA methylation. Baca SC et al. developed a proof-of-concept comprehensive epigenomic profiling method with plasma samples from advanced cancers, using an immunoprecipitation-based approach targeting histone modifications and DNA methylation to capture the dynamic biology of tumors [176]. This technique effectively differentiated between various cancer subtypes, such as prostate adenocarcinoma and NEPC. Additionally, it allowed for real-time monitoring of changes in tumor phenotypes while providing valuable insights that complement genetic analyses. In the previous section, we highlighted a study that utilized epigenomic profiling to identify aggressive signatures in advanced prostate cancer [56]. The clinical utility of plasma epigenomic profiling was recently illustrated in a case report that showcased the first use of cell-free DNA chromatin immunoprecipitation sequencing (cfCHIP-seq) to non-invasively detect a rare resistance mechanism in advanced prostate cancer [177]. The patient was initially diagnosed with de novo metastatic prostate adenocarcinoma, but later experienced disease progression while on 177Lu-PSMA-617 therapy, leading to the emergence of treatment-related squamous cell prostate cancer. Notably, the cfChIP-seq liquid biopsy identified molecular signatures of squamous cell prostate cancer in the patient’s plasma more than nine months prior to clinical diagnosis through biopsy, highlighting the potential of epigenomic assays to detect lineage plasticity and resistance phenotypes.

Silva et al. conducted a study on urine and blood samples from four metastatic treatment-naïve prostate cancer patients, focusing on DNA methylation profiling with the Infinium^®^ MethylationEPIC BeadChip [178]. The DNA methylation profiles from both urine and plasma showed a strong correlation (ρ = 0.93), with a significant contribution from non-tumor sources. While the sensitivity of detection varies, plasma was able to identify up to 64% and urine up to 39% of tumor-specific methylation alterations, although these findings differed among patients.

Enhancer of Zeste Homolog 2 (EZH2) is a histone methyltransferase that functions as the catalytic subunit of the Polycomb Repressive Complex 2 and is responsible for regulating the epigenetic mark H3K27, which is essential for transcriptional repression and chromatin condensation [179,180]. In mCRPC and NEPC, EZH2 is often overexpressed, playing a pivotal role in disease progression, metastasis, and resistance to therapies targeting the androgen receptor [179]. Recent studies using plasma epigenomic profiling methods, such as cfChIP-seq and cfDNA methylation, have demonstrated a non-invasive approach for monitoring the regulation of the EZH2 gene in advanced prostate cancer [176,179,181]. This innovative strategy could enable the early detection of lineage plasticity and the transition to NEPC. Furthermore, the EZH2 epigenetic driver may serve as a valuable predictive biomarker for clinical trials exploring novel combination therapies, including EZH2 inhibitors like tazemetostat and valemetostat [182,183].

## 12. Challenges and Limitations with ctDNA

Despite the vast impact of liquid biopsy, several limitations affect its clinical validation and routine application. These restrictions span the entire diagnostic workflow, from pre-analytical handling to post-analytical result interpretation, and often derive from the lack of protocol standardization [184]. In 2018, an external quality assessment (EQA) revealed substantial variability in cfDNA diagnostic methodologies across 42 institutions in 10 European countries, with an overall genotyping error rate of 6.1% [185]. In the pre-analytical phase, a major issue is the contamination of plasma with genomic DNA released by leukocyte lysis during blood collection [186]. Standard EDTA blood collection tubes (BCTs) must be processed within 6 h to minimize cell lysis, which otherwise increases background noise by releasing wild-type molecules, thus diluting the cfDNA fraction. To mitigate this risk, BCTs with stabilizing fixatives (e.g., Streck cfDNA BCTs, PAXgene) have been developed [187,188]. However, widespread commercialization has outpaced head-to-head validation studies that could identify emergent issues not otherwise anticipated, including chemical modifications that interfere with downstream analyses, such as potential cross-linking that compromises methylation profiling [189]. Another critical pre-analytical challenge concerns cfDNA extraction, which is based on either manual (i.e., silica-based spin columns) or automated strategies (i.e., magnetic bead-based workflows). Automated bead systems are widely adopted because they generally recover short cfDNA fragments more efficiently, whereas many spin-column kits are designed for high-molecular-weight genomic DNA [190]. However, prioritizing short fragments can miss longer DNA fragments harboring crucial alterations possibly deriving from necrotic tissues, underscoring the need for strategies that capture a broader fragment spectrum. Indeed, the European DNA-Plas study reported that, across 56 laboratories, only 12.5% were able to amplify >400 bp fragments after extraction [191].

From an analytical perspective, cfDNA testing faces mainly sensitivity constraints. Sensitivity is limited by false negatives in low-shedding contexts, where ctDNA concentrations fall below the assay limit of detection. In prostate cancer, TFxs are higher in mCRPC than in mHSPC or localized disease [84,192]. However, undetectable cfDNA levels also occur in mCRPC with less aggressive presentations, such as limited skeletal burden (i.e., ≤10 bone metastases), absence of liver metastases, and node-only disease [21].

Additionally, para-physiological conditions may increase total cfDNA levels, further diluting the malignant DNA fraction and reducing the sensibility of the assays. For instance, physical exercise induces a rise in cfDNA released from polymorphonuclear leukocytes and cardiomyocytes, facilitated by increased body temperature and reduced oxygen availability [193]. Similarly, increased cfDNA release can also rise during inflammatory and infectious states [194,195]. By contrast, specificity is generally high due to error-correction strategies (e.g., UMIs) that suppress background artifacts at very low VAFs. However, an important concern remains clonal hematopoiesis (CH) of indeterminate potential (CHIP), an age-associated process in which hematopoietic stem cells acquire somatic mutations that confer a selective advantage, leading to clonal expansion [196]. Since these alterations arise in blood cells, they can appear as false positives in cfDNA, leading to misdiagnosis [197]. Its prevalence rises with age, from 20% between 65 and 69 years to 50% at ≥80 years [198]. The most frequently involved genes are epigenetic regulators (i.e., *DNMT3A*, *TET2*, *ASXL1*), although the alterations of greater clinical impact affect DNA-damage response (DDR) genes such as *TP53*, *CHEK2*, and *BRCA1/2* [199]. Jensen et al. tested 69 men with advanced prostate cancer, finding CH variants in 13 (19%) cases, seven of whom (10%) harbored DDR mutations potentially targetable with PARPI (*ATM n* = 5, *BRCA2 n* = 1, *CHEK2 n* = 1) [200]. By analyzing cfDNA across 49 tumor types, other authors reported that among somatic alterations, 76.0% of *CHEK2*, 58.6% of *BRCA1*, and 46.2% of *ATM* variants were of CH origin [198]. To overcome the CHIP phenomenon, the gold standard remains deep sequencing of leukocyte DNA, as recommended by the European Society of Medical Oncology precision medicine working group [145]. However, this approach requires a larger starting material, which can be challenging in cases with limited samples [201]. Emerging alternatives for CHIP diagnostics include algorithmic subtraction [198], fragmentomics-based filtering [202], and plasma-only machine learning frameworks (e.g., MetaCH) [203], but all require further validation.

In the post-analytic phase, the key challenge is to condense all prior steps into a clear and easily reproducible and interpretable analysis. First, the report should concisely disclose sample collection, preparation, and storage methods, together with assay performance metrics (e.g., depth/coverage, VAFs, LODs) [184]. This point hinders standardization efforts, since methods remain heterogeneous and poorly harmonized across different laboratories and vendors. A review of 1228 ctDNA publications showed inconsistent disclosure of pre-analytical variables and performance metrics: only 30% reported analytical specificity and 47% reported assay sensitivity; among NGS studies, 73% omitted minimum coverage and 51% omitted mean coverage [204]. Another challenging step for interpretability is variant actionability classification. Beyond adopting widely accepted grading systems (e.g., AMP tiers, ESCAT) supported by high-quality evidence and databases (e.g., OncoKB, COSMIC, ClinVar/CIViC), a clearer approach to addressing confounders is needed [184]. For instance, a major implementation of orthogonal confirmatory tests for validating cfDNA results is still lacking. The literature revision conducted by the Liquid Biopsy Working Group (LBxWG) of the Association for Molecular Pathology (AMP), ASCO and the College of American Pathologists (CAP) highlighted that only 11% of the analyzed publications reported using orthogonal or confirmatory methods [204]. Standardization and scalability require coordinated processes, with the goal of reducing costs. Strengthening the establishment of shared international platforms that openly disseminate protocols and curate up-to-date, centralized databases of reagents and products selected through methodologically transparent studies would increase the quality of subsequent research and ensure greater reliability across the multitude of assays on the market. Furthermore, greater investment in quality-assessment initiatives is needed, building on efforts by international consortia such as CANCER-ID in Europe (https://www.ihi.europa.eu/projects-results/project-factsheets/cancer-id (accessed on 28 April 2026)) and BloodPAC in the United States (www.bloodpac.org) [205]. In addition, the absence of defined clinical purpose at the assay-design stage hampers appropriate allocation in subsequent practice settings. Closer collaboration between clinicians and developers would help reduce costs by identifying the optimal goal for more or less expensive technologies (e.g., deep-coverage NGS versus digital PCR). Long-term cost-effectiveness analyses are urgently needed, as illustrated by the VALUE trial, where higher upfront costs for liquid biopsy were offset by those patients receiving matched targeted therapies, thus avoiding expenses for non-personalized and potentially futile therapies [206].

## 13. Future Directions and Conclusions

In the current clinical landscape for metastatic prostate cancer, established guidelines recommend using ctDNA as an option for targeted therapy selection [7,207]. ctDNA has proven effective in identifying HRR alterations, with emerging evidence also supporting its use for detecting MSI-H or TMB-H status to guide immune checkpoint inhibitor therapy (Table 2). ctDNA fraction is a strong prognostic factor and an effective tool for monitoring response to systemic treatment (Table 1), outperforming PSA and other clinical factors in several prostate cancer studies. Additionally, ctDNA clearance has consistently emerged as a robust predictor of improved clinical outcomes, paving the way for potential opportunities to serve as an early endpoint in clinical trials. The RECIST working group has evaluated the role of ctDNA in metastatic cancer clinical trials and currently recommends its use primarily for exploratory purposes or as secondary endpoints within these trials [208]. Moreover, ctDNA-guided precision medicine approaches in advanced prostate cancer enable therapy selection based on genomic alterations identified on liquid biopsy. Currently, the CCTG is conducting a clinical trial (NCT03385655) that utilizes ctDNA to match patients with mCRPC with targeted therapies informed by specific genomic biomarkers [160,209]. The emergence of resistance to ARPIs due to alterations in the androgen receptor (AR) is common and is associated with poor clinical outcomes. ctDNA has proven to be an effective non-invasive method for identifying these alterations under genomic pressure within AR pathways (Table 2). ctDNA analysis provides valuable insights into the AR mutational profile, offering potential for next-generation AR degraders investigations. These novel hormonal therapies bind to the AR and recruit E3 ubiquitin ligases, triggering the ubiquitination of AR and its subsequent proteasomal destruction, thereby suppressing both wild-type and mutant AR-driven prostate cancers more effectively than traditional antagonists. Currently, there are multiple ongoing clinical trials (NCT05067140, NCT04428788, NCT05252364, NCT06764485) focused on mCRPC, particularly in tumors that harbor ligand-binding domain mutations or amplifications. EZH2 plays a crucial role in resistance and lineage changes in mCRPC and is considered a potential target for therapy. Although ctDNA assays can detect EZH2 alterations, its expression and related signaling pathways are not yet routinely evaluated in clinical practice and its clinical role remains investigational. However, several clinical trials are currently exploring EZH2 inhibitors in combination with ARPIs or PARPIs for mCRPC (NCT04179864, NCT03460977, NCT06551324, NCT04846478, and NCT06629779).

Importantly, the advancement of ctDNA-based strategies in precision oncology requires careful consideration of the impact of racial disparities on genomic analyses. Data from the AACR Project GENIE database have shown that Black men with metastatic prostate cancer harbor higher frequencies of AR mutations (18.3% vs. 8.1%, *p* = 0.004), DDR alterations (22.5% vs. 15.6%, *p* = 0.05), and actionable genomic alterations (26.7% vs. 18.0%, *p* = 0.05) compared with White men [210]. A large ctDNA-based study confirmed these findings, reporting that AR alterations were significantly more frequent in Black than in White men (55.3% vs. 35.0%, *p* < 0.001) [211]. However, landmark molecular profiling studies such as the Cancer Genome Atlas (TCGA) prostate cancer cohort have included only a limited proportion of African American patients (10–13%) [212,213]. Efforts should therefore focus on addressing these disparities to ensure a more comprehensive representation of the molecular landscape of prostate cancer.

As the field of prostate cancer advances, the integration of machine learning and artificial intelligence (AI) tools presents a promising opportunity to enhance the development of liquid biopsies for advanced prostate cancer. AI algorithms can be leveraged to enhance the accuracy of ctDNA mutational profiling, improve the sensitivity of ctDNA detection, and clarify the interpretation of complex genomic and transcriptomic patterns. AI-enabled whole-genomic sequencing platforms and multi-omic platforms possess the potential to revolutionize early detection and the monitoring of minimal residual disease [214,215], thereby overcoming the limitations of currently available clinical ctDNA assays.

In conclusion, liquid biopsies have emerged as pivotal biomarkers in the management of prostate cancer, representing an essential tool for both prognostic and predictive assessment and for guiding personalized therapy selection.

## Figures and Tables

**Table 1:** Summary of ctDNA studies in advanced prostate cancer with prognostic significance.

Studies	Design	N	ctDNA Assay	Systemic Tx	Timing of Sampling	Prognostic Impact of Genomic Alterations	Prognostic Impact of ctDNA Fraction Dynamics
Kohli M et al. [84]	Longitudinal prospective cohort study	250 (NGS analyzable). mHSPC (untreated *n* = 73, on ADT *n* = 33); mCRPC (biochemical *n* = 75, clinical *n* = 69). Includes high- and low-volume mHSPC	120-gene PredicineLDT hybrid-capture panel, with matched germline DNA	ADT	Baseline, 3 mo post ADT, and at progression	Untreated mHSPC: DDR gene alterations (*ATM*, *BRCA1/2*, *CHEK2*) shorten OS and ADT failure time mCRPC: AR gain and *TP53* mutations correlate with poorer OS	High-volume disease with high ctDNA TFx associated with the worst outcomes, while low-volume disease with low ctDNA TFx shows the best results. High-volume disease with low ctDNA TFx has intermediate outcomes
Yang B et al. [91]	Retrospective multicenter Chinese cohort study	182, HSPC (localized [*n* = 24], lymph node disease [*n* = 44], low-volume mHSPC [*n* = 42], high-volume mHSPC [*n* = 72])	The custom, hybrid-capture next-generation sequencing panels run at Glorious MeClinical Laboratory	ADT +/− Abiraterone/chemotherapy	71% treatment-naïve at ctDNA sampling; 29.1% had prior systemic therapy	*BRCA1/2*, *TP53*, *PTEN*, *RB1,* or *CDK12* associated with shorter time to CRPC in high-volume mHSPC	NR
Shiota M et al. [83]	Prospective observational study	163, mHSPC (*n* = 68) and mCRPC (*n* = 95)	FoundationOne Liquid CDx	ADT, ARPI, Taxanes	Pre-treatment and post-progression	HRR alterations in mHSPC are associated with shorter time to CRPC in ADT group. AR amplification/mutation associated with shorter time to treatment failure in mCRPC	ctDNA TFx 10% in mHSPC is associated with shorter time to CRPC
Lee CU et al. [94]	Prospective study	75, mHSPC	Low-depth WGS of plasma cfDNA	ADT	Before ADT initiation	cfDNA-based chromosomal instability (CIN). Shorted time to progression on ADT and higher risk of progression to mCRPC strongly linked to High CIN Mutations not reported in this study	NA
Vandekerkhove G et al. [63]	Prospective study	53, De novo mHSPC	Targeted sequencing (73 prostate cancer driver genes)	ADT, ADT + Docetaxel or ADT + ARPI	Before and after ADT initiation	*TP53* mutations, DDR gene alterations associated with earlier progression to CRPC	NR This study examined the feasibility and complementarity of ctDNA compared to tissue samples
Fonseca NM et al. [21]	Meta-analysis of prospective trials and biobanking	491, mCRPC, 738 plasma cfDNA samples	A custom, research-grade hybrid-capture targeted NGS assay on plasma cfDNA with matched WBC sequencing	Abiraterone, Enzalutamide and Taxanes (Docetaxel, Cabazitaxel)	Prior to 1st-, 2nd-, or 3rd-line therapy	NR	ctDNA% strongly prognostic for OS, clinical/r PFS across treatment lines. High baseline ctDNA% (>30%) linked to 5.6× greater risk of death vs. <2%
Crippa A et al. [95]	Prospective randomized platform trial (substudy)	220, mCRPC (139 with detectable ctDNA, 81 with undetectable ctDNA)	Hybridization-based capture (prostate-specific design)	ARPI or Taxanes	Baseline before treatment	NR	Undetectable ctDNA levels at baseline predict a better prognosis, regardless of therapy. Each 10% increase in ctDNA tumor fraction corresponds to a 10% reduction in survival time
Sweeney CJ et al. [96]	Retrospective analysis of a phase III trial (IMbassador250)	494, mCRPC (post-Abiraterone)	FoundationOne Monitor (tissue-naïve assay)	Enzalutamide +/− immunotherapy	Baseline and cycle 3 day 1 (C3D1)	NA	Detection of ctDNA TFx at C3D1 associated with worse rPFS and OS. Discordant cases (undetected TFx but no PSA reduction) had favorable outcomes
Annala M et al. [97]	Prospective randomized phase II trial	95, mCRPC with poor prognosis features	Research-grade deep targeted sequencing (73-gene assay)	Cabazitaxel or Abiraterone, Enzalutamide	Baseline, cycle 4, and progression	*RB1*, *TP53*, and AR alterations enriched in poor prognosis patients. AR amplification associated with shorter OS and TTP	Baseline ctDNA TFx > 30% had markedly shorter OS than undetectable ctDNA. On treatment ctDNA increase linked to shorter TTP
Annala M et al. [98]	Prospective randomized phase II study	202, mCRPC (treatment-naïve)	Whole-exome and targeted 72-gene sequencing	Abiraterone, Enzalutamide	Baseline prior to systemic therapy	*BRCA2*, *ATM*, and *TP53* are strongly associated with poor outcomes and primary resistance. AR structural rearrangements associated with primary resistance	ctDNA TFx > 30% associated with poor therapy response, TTP independent of clinical factors
Fettke H et al. [99]	Prospective registry study	150, mCRPC	Customized 78-gene panel	Lu-PSMA (177Lu-PSMA-617)	Baseline, week 6 (cycle 2), week 18 (cycle 4), and progression	*AR* and *TP53* were frequent alterations. FOLH1 alterations associated with resistance. WNT pathway alterations associated with shorter PSA-PFS	Baseline ctDNA TFx is an independent prognostic factor for OS. Undetectable ctDNA at week 6 linked to superior benefit
Zhao Y et al. [100]	Longitudinal prospective cohort study	60, mCRPC (277 serial samples)	Sparse whole-genome sequencing and deep whole-exome sequencing	ARPI (Abiraterone, Enzalutamide), Taxanes (Docetaxel, Cabazitaxel), and Radium-223	Before, during, and upon progression	ARPI resistance associated with *ZFHX3*, *FANCA*, *RB1*, *PIK3CB*, *PTEN*, *BRCA2*, and AR aberrations. Taxane resistance linked to CTCF (Chr16q22.1) alterations and PTEN loss, while *TP53* mutations are often acquired	Higher ctDNA TFx (>13.7%) at baseline associated with worse OS. Evolutionary Dynamic Index low levels associated with poor survival
Goodall J et al. [45]	Prospective phase II trial (TOPARP-A)	50, mCRPC (46 for cfDNA analysis)	Targeted sequencing (custom panel) and whole-exome sequencing	Olaparib	Baseline, weeks 1, 4, 8, and 16, and progression	Emergence of second-hit reversion mutations in *BRCA2* and *PALB2* due to Olaparib at progression	A 50% decrease in cfDNA concentration after 8 weeks independently associated with longer OS (HR 0.19)
Shaya J et al. [101]	Retrospective study	63, mCRPC	Commercial assays (Guardant 360 or Tempus xF)	ARSI, Taxanes	Heterogeneous sample collection (mostly during progression)	More than one alteration is linked to worse OS compared to 0–1 alterations. Actionable alterations with FDA-approved options for HRR or MMR genes were found in 16% of patients	Maximum allelic fraction (mAF) > 6.4% associated with inferior OS (8 vs. 25 mo, HR 3.9), though mAF lost significance in multivariable models
Knutson TP [102]	Prospective (Alliance A031201 phase 3 trial)	776, mCRPC	AR-ctDETECT (custom targeted DNA-seq) and ichorCNA	Enzalutamide +/− Abiraterone	Baseline, single pre-treatment timepoint	ctDNA-positive status associated with worse OS (27 vs. 47 mo, HR 2.0) and rPFS (18 vs. 33 mo). *TP53*, *PTEN*, and *RB1* mutations or copy number loss were common in ctDNA-positive patients, especially those with high aneuploidy, and associated with poorer prognosis	Higher ctDNA TFx (ctDNA aneuploidy fraction) was a strong prognostic marker for worse outcomes
Antonarakis ES et al. [103]	Retrospective (real-world database analysis)	8420, mCRPC	Commercial assay (Guardant360)	Abiraterone, Enzalutamide, Apalutamide, Darolutamide	ctDNA was not sampled at predetermined protocol-defined times	AR-LBD mutation prevalence increases with treatment lines (15% to 24%). AR-LBD+ patients had shorter OS (50 vs. 61 mo, *p* = 0.013). High enrichment of HRR, PI3K, and RB1 mutations in AR-LBD+ cohort	NR AR-LBD+ cohort had higher mean ctDNA burden (VAF 18.94% vs. 10.04%)
Tripathi N et al. [104]	Retrospective	137, mCRPC	Commercial assay (Guardant360 or Tempus xT)	Abiraterone/Enzalutamide	cfDNA sampling either before or after initiation of the first-line ARPI	AR alterations associated with significantly inferior OS post-progression on first ARPI (15 vs. 30 mo, *p* < 0.001). Taxane efficacy independent of AR status	NR
Kwan EM et al. [105]	Prospective	231, mCRPC	Predicine targeted NGS assay	ARPI or Taxane	Baseline	PTEN loss (37%) and AR gain (42%) independently confer poor OS. Combined AR gain + PTEN loss + PIK3CA gain associated with highest risk (HR 3.2). Cumulative CNV burden (PTEN/PI3K/AR) significantly associated with worse OS	Higher ctDNA TFx associated with worse clinical outcomes

ADT: androgen deprivation therapy, AR: androgen receptor, ARPI: androgen receptor pathway inhibitor, cfDNA: cell-free DNA, CNV: copy number variations, DDR: DNA damage repair, HR: hazard ratio, HRR: homologous recombination repair, mHSPC: metastatic hormone-sensitive prostate cancer, mCRPC: metastatic castration-resistant prostate cancer, mo: months NA: not available, NGS: next-generation sequencing, NR: not reported, OS: overall survival; PSA: prostate-specific antigen, PSMA: prostate-specific membrane antigen, TFx: tumor fraction, TTP: time to progression.

**Table 2:** Summary of ctDNA studies in advanced prostate cancer with predictive value.

Studies	Design	N	Platform/Assay for ctDNA Analysis	Systemic Tx	Timing of Sampling	Predictive Impact of Genomic Alterations	Predictive Impact of ctDNA Fraction Dynamics	Comments
Du X et al. [93]	Retrospective study	66; (mHSPC low volume 80%, high volume 20%)	In-house targeted gene NGS panel	ADT plus Docetaxel	Baseline (pre-treatment) and a second sample after 1 cycle of treatment	De novo HRR pathway alterations (*ATM*, *BRCA2*) after treatment associated with shorter time to castration resistance (8 vs. 13 mo) and worse clinical outcome	Elevated ctDNA fraction after 1 cycle of chemotherapy predicts shorter time to castration resistance (8 vs. 18 mo, *p* < 0.001)	Early serial ctDNA monitoring provides predictive value for chemohormonal therapy efficacy in mHSPC
Herberts C et al. [115]	Retrospective study	599; mCRPC	Targeted cfDNA sequencing (custom panel)	Abiraterone, Enzalutamide, Docetaxel	Baseline and serial. Sampling not at a single uniform timepoint	*AKT1/PIK3CA* mutations (6% prevalence) delineate subtype with low AR copy gain. OS and PSA-PFS similar to wild-type. AKT1 mutations are mutually exclusive with PTEN alterations	NR	Clonal mutations were consistent across serial ctDNA collections. *AKT1/PIK3CA* mutation fraction correlates with ipatasertib response in case report
Torquato S et al. [116]	Prospective study	62; mCRPC	Deep NGS (46 genes)	Enzalutamide, Abiraterone	Baseline and at progression	*AR* LBD mutations are associated with shorter PFS (HR 2.51, *p* = 0.020). *TP53* alterations associated with worse OS (HR 2.70, *p* = 0.009). PI3K pathway alterations associated with worse OS	High baseline ctDNA TFx and rising TFx over time associated with resistance, poor benefit with ARPI and worse clinical outcomes (PFS, OS)	*TP53* and *RB1* alterations together were associated with significantly worse OS (HR 4.56). Lower or stable ctDNA TFx associated with better responses
De Bono et al. [117]	Prospective phase 3 study (PSMA-fore)	468 (156 with ctDNA >1%)	Targeted NGS (585 genes)	177Lu-PSMA-617 vs. ARPI change (Taxane-naïve mCRPC)	Baseline and early in treatment	Presence of baseline 8q amplifications, AR amplifications, and TP53 deleterious alterations were associated with shorter rPFS and decreased tumor response in the 177Lu-PSMA-617 arm	Early ctDNA clearance was strongly associated with longer rPFS and improved tumor response	Baseline ctDNA fraction >1% adversely associated with rPFS, RECIST, and PSA50 response across both arms. 177Lu-PSMA-617 prolonged rPFS regardless of baseline ctDNA
Azad et al. [88]	Prospective phase 3 study (TALAPRO-2)	678	FoundationOne Liquid CDx	Talazoparib + Enzalutamide vs. placebo + Enzalutamide (1st-line mCRPC)	Baseline and week 9	Not reported	Conversion from high-to-low at W9 prognostic of improved rPFS vs. remaining high (Talazoparib: 16.6 vs. 5.5 mo; placebo: 10.9 vs. 2.6 mo). Remaining low showed greater rPFS benefit vs. high-to-low conversion (Talazoparib HR = 0.45, *p* = 0.0003; placebo HR = 0.34, *p* < 0.0001)	High baseline ctDNA burden was adversely prognostic for rPFS across both arms. Study was limited by the prototype tumor fraction algorithm’s limit of quantification
De Giorgi U et al. [112]	Prospective phase 2 study	40; mCRPC	Digital droplet PCR	Lu-PSMA (177Lu-PSMA-617)	Within 28 days of treatment initiation	AR gene gain/amplification associated with early progression and shorter OS and PFS	NA	Patients with no AR alterations showed durable benefit to Lu-PSMA
Halabi S et al. [114]	Prospective (Alliance A031201 phase 3 trial)	776; mCRPC	AR-ctDETECT assay (targeted ctDNA sequencing)	Enzalutamide +/− Abiraterone	Baseline, single pre-treatment timepoint	PGAs (gains in AR, AR enhancer, MYC; losses in PTEN, *TP53*, *RB1*, NKX3–1) associated with poorer OS (27 vs. 47 mo). Clinical genetic model improved OS prediction accuracy	NA	ctDNA-derived PGAs enhance OS predictions by 30% and correlate with poorer clinical variables
Ravindranathan D et al. [118]	Retrospective case series	2; mCRPC	Commercial assay (Guardant360)	Pembrolizumab	Baseline and serial	ctDNA detected MSI-H status. Both cases showed excellent response	NR	Repeat ctDNA showed complete clearance of somatic alterations and MSI-H status in Case 1, and 15% to 0.5% reduction in Case 2
Barata P et al. [119]	Retrospective case series	9; mCRPC;	Commercial assay (Guardant360)	Pembrolizumab	Baseline ctDNA for clinical decision-making	MSI-H predicted robust response: 44% PSA50 response rate Co-occurring genomic alterations include *TP53*, *AR*, *BRCA1/2*, *PIK3CA*, *ATM*. Responses associated with combined MSI-H and DDR gene alterations	NA ctDNA was used to detect MSI-H and co-occurring alterations rather than to track ctDNA dynamics over time	ctDNA is feasible to identify MSI-H status where tissue biopsy is difficult
Azad AA et al. [120]	Retrospective	62; mCRPC	Array comparative genomic hybridization and Roche 454 NGS	Abiraterone, Enzalutamide	Baseline, at progression	AR amplification linked to Enzalutamide resistance (53% in progressors) AR aberrations in pre-treatment cfDNA associated with lower PSA response rates and shorter PFS	NR	cfDNA shows high concordance with metastatic tumor biopsies for AR status and indicates therapeutic resistance
Sumiyoshi T et al. [121]	Prospective/retrospective	102; mCRPC (Japanese patients)	dPCR and target sequencing	Abiraterone, Enzalutamide, Taxanes	Baseline and serial	AR aberrations (gain/mutations L702H, T878A, H875Y) associated with poor response to Abiraterone (median PSA-PFS 67 vs. 342 days), but not Enzalutamide	NR	Baseline AR alterations diminished with effective treatment; new AR amplifications/mutations (T878A, L702H, H875Y) emerged at progression
Wyatt AW et al. [122]	Temporal observation cohort	65; mCRPC	aCGH and deep AR exon 2–8 sequencing (Illumina MiSeq)	Enzalutamide	Baseline, 12 weeks, and at progression	AR amplification, heavily mutated AR (≥2 mutations) and RB1 loss associated with worse PFS. *TP53* and *CTNNB1* mutations identified at progression	NR	Clonal selection observed during treatment; emergence of AR L702H, H875Y and T878A in patients with prior Abiraterone/prednisone
Jayaram A et al. [123]	Pooled analysis of prospective cohorts	501; mCRPC	ddPCR and targeted capture NGS	Abiraterone or Enzalutamide	Single baseline (before first-line ARPI)	AR copy number (CN) ≥1.92 identifies aggressive disease with shorter OS and shorter PFS. AR gain associated with shorter prior response to primary ADT	NA	AR CN gain correlates with increased total cfDNA yield and tumor volume markers but is used unadjusted for TFx in clinical dichotomization
Rathkopf DE et al. [124]	Phase I/II study	93, (51 nmCRPC, 46 mCRPC)	BEAMing (digital PCR)	Apalutamide	Baseline before starting Apalutamide and at progression	AR T878A associated with Abiraterone resistance Acquisition of AR F877L at progression in 3.7% of patients. Decrease/loss of T878A mutation in 2 of 3 post-Abiraterone patients on Apalutamide	NR	AR-LBD mutations like F877L and T878A are not common contributors to Apalutamide resistance
Kwan EM et al. [87]	Post hoc biomarker analysis of a randomized phase II trial (TheraP)	180, mCRPC (biomarker population, *n* = 178)	Custom targeted panel (76 genes + whole-genome backbone)	Lu-PSMA (Lutetium-177-PSMA-617) vs. Cabazitaxel	Baseline (pre-treatment) and at progression	PTEN alterations with worse outcomes while on Cabazitaxel. ATM and BRCA2 defects are found in exceptional responders, while TP53 is linked to reduced survival, and AR status is non-predictive	NR	PSMA-PET (SUVmean) and ctDNA% were independent predictors; ctDNA TFx < 2% predicts better outcomes on Lu-PSMA and overall survival
José C. Valentín López et al. [125]	Prospective (secondary analysis of Alliance A031201 phase 3 trial)	327; mCRPC	AR-ctDETECT targeted cfDNA sequencing assay (820 kb across 69 genes)	Enzalutamide or Enzalutamide + Abiraterone	Baseline and at radiographic progression	Non-AR alterations (*TP53*, *PTEN*, *RB1*, *FANCA*) indicate primary resistance in rapid progressors (rPFS < 6 mo), while AR alterations (copy gains, GSRs) are more common in delayed progressors (rPFS > 30 mo)	NR	Identified an AR extrachromosomal DNA signature (AR gain + 2+ GSRs) that promotes AR structural diversification and LBD-truncating variants during treatment resistance

ADT: androgen deprivation therapy, AR: androgen receptor, ARPI: androgen receptor pathway inhibitor, cfDNA: cell-free DNA, ctDNA: circulating tumor DNA, DDR: DNA damage repair, GSR: genomic structural rearrangement, HR: hazard ratio, HRR: homologous recombination repair, LBD: ligand-binding domain, mCRPC: metastatic castration-resistant prostate cancer, mo: months, mHSPC: metastatic hormone-sensitive prostate cancer, MSI-H: microsatellite instability-high, NA: not available, NGS: next-generation sequencing, NR: not reported, OS: overall survival; PGAs: pathogenic genetic alterations, PCR: polymerase chain reaction, PSA: prostate-specific antigen, PSMA: prostate-specific membrane antigen, TFx: tumor fraction.

## Data Availability

No new data were created or analyzed in this study.

## Abbreviations

ADT	Androgen deprivation therapy
AR	Androgen receptor
ARPIs	Androgen receptor pathway inhibitors
AR-V7	Androgen receptor splice variant 7
BCTs	Blood collection tubes
BEAMing	Beads, Emulsion, Amplification, and Magnetics
CAPP-Seq	Cancer Personalized Profiling by deep Sequencing
cfChIP-seq	cell-free chromatin immunoprecipitation sequencing
cfDNA	cell-free DNA
CH	Clonal hematopoiesis
CHIP	Clonal hematopoiesis of indeterminate potential
CIN	Chromosomal instability
CNAs	Copy number alterations
CNV	Copy number variation
CRPC-NE	Castration-resistant prostate cancer with neuroendocrine phenotype
CT	Computed tomography
CTC	Circulating tumor cell
ctDNA	circulating tumor DNA
ddPCR	droplet digital PCR
DDR	DNA damage response
dMMR	mismatch repair deficiency
EDI	Evolutionary dynamic index
EZH2	Enhancer of zeste homolog 2
GWA	Genome-wide aneuploidy score
HRR	homologous recombination repair
LBD	Ligand-binding domain
LDH	Lactate dehydrogenase
mCRPC	metastatic castration-resistant prostate cancer
mFAST-SeqS	modified Fast Aneuploidy Screening Test-Sequencing
mHSPC	metastatic hormone-sensitive prostate cancer
MRD	minimal residual disease
MSI-H	microsatellite instability-high
NEMO	Neuroendocrine detection and monitoring
NEPC	Neuroendocrine prostate cancer
NGS	Next-generation sequencing
OS	overall survival
PARP	Poly(ADP-ribose) polymerase
PARPi	PARP inhibitors
PCR	polymerase chain reaction
PDX	Patient-derived xenograft
PET	Positron emission tomography
PFS	progression-free survival
PSA	Prostate-specific antigen
PSMA	Prostate-specific membrane antigen
qPCR	quantitative PCR
rPFS	radiographic progression-free survival
TEC-Seq	Targeted Error Correction Sequencing
TFx	tumor fraction
TMB	tumor mutational burden
UMIs	unique molecular identifiers
VAF	variant allele frequency
WES	whole-exome sequencing
WGS	whole-genome sequencing
